# Sex differences in DNA methylation assessed by 450 K BeadChip in newborns

**DOI:** 10.1186/s12864-015-2034-y

**Published:** 2015-11-09

**Authors:** Paul Yousefi, Karen Huen, Veronica Davé, Lisa Barcellos, Brenda Eskenazi, Nina Holland

**Affiliations:** School of Public Health, University of California, 733 University Hall, School of Public Health, UC, Berkeley, CA 94720-7360 USA

**Keywords:** Epigenetics, Gene ontology, Birth cohort, EWAS, Gender, Cord blood

## Abstract

**Background:**

DNA methylation is an important epigenetic mark that can potentially link early life exposures to adverse health outcomes later in life. Host factors like sex and age strongly influence biological variation of DNA methylation, but characterization of these relationships is still limited, particularly in young children.

**Methods:**

In a sample of 111 Mexican-American subjects (58 girls , 53 boys), we interrogated DNA methylation differences by sex at birth using the 450 K BeadChip in umbilical cord blood specimens, adjusting for cell composition.

**Results:**

We observed that ~3 % of CpG sites were differentially methylated between girls and boys at birth (FDR *P* < 0.05). Of those CpGs, 3031 were located on autosomes, and 82.8 % of those were hypermethylated in girls compared to boys. Beyond individual CpGs, we found 3604 sex-associated differentially methylated regions (DMRs) where the majority (75.8 %) had higher methylation in girls. Using pathway analysis, we found that sex-associated autosomal CpGs were significantly enriched for gene ontology terms related to nervous system development and behavior. Among hits in our study, 35.9 % had been previously reported as sex-associated CpG sites in other published human studies. Further, for replicated hits, the direction of the association with methylation was highly concordant (98.5–100 %) with previous studies.

**Conclusions:**

To our knowledge, this is the first reported epigenome-wide analysis by sex at birth that examined DMRs and adjusted for confounding by cell composition. We confirmed previously reported trends that methylation profiles are sex-specific even in autosomal genes, and also identified novel sex-associated CpGs in our methylome-wide analysis immediately after birth, a critical yet relatively unstudied developmental window.

**Electronic supplementary material:**

The online version of this article (doi:10.1186/s12864-015-2034-y) contains supplementary material, which is available to authorized users.

## Background

There is a growing interest in examining the role epigenetic marks like histone modifications, non- coding RNAs, and DNA methylation may play as biological mechanisms through which environmental exposures and other physiological and lifestyle factors can lead to disease. Unlike genetics, epigenetic modifications are dynamic and can change over time or in response to exposures. Furthermore, host factors such as sex and age also contribute to inter-individual differences in epigenetic markers.

Previous studies of DNA methylation using the Illumina 27 K BeadChip methylation array have reported autosomal differentially methylated positions (DMPs) or CpG sites with varying methylation between males and females, providing evidence that it will be important to adjust for sex in analysis of methylation data [1–6]. However, these studies did not account for the existence of non-specific probes for autosomal CpGs that cross react with CpGs on sex chromosomes, thereby yielding false positives [7]. Recently, McCarthy et al. published a meta-analysis of 76 studies all using the 27 K BeadChip array to identify sex-associated autosomal DMPs across specimens from multiple tissue types from adults and children [8]. After excluding the sex-biased cross-reactive probes, they identified 184 DMPs that were associated with sex.

While McCarthy et al. identified several interesting autosomal DMPs, their study focused on methylation assessed by the 27 K BeadChip. In 2011, Illumina released a new version of their methylation array, the 450 K BeadChip, which greatly expanded the number of CpGs interrogated to over 480,000 sites. Further, their approach was restricted to identification of individual DMPs rather than differentially methylated regions (DMRs). DMR-finding approaches have several advantages over considering CpG sites individually, including decreased likelihood of hits from technical artifacts and possibly improved functional impact of results.

As methylation is cell-type specific and immune cell profiles have been shown to vary between sexes, consideration of cell composition is of utmost importance in methylation studies [9, 10]. Yet previous studies of sex-associated differences in methylation [1–6] haven’t taken this into account in their analyses. White blood cell composition can be estimated from 450 K BeadChip data computationally in adults [11, 12], but these estimates are not appropriate for use for young children in their current implementation [13]. As an alternative, differential cell count (DCC) can be employed to effectively determine such cell type proportions (% lymphocytes, monocytes, neutrophils, eosinophils, and basophils) in cord blood samples.

Here, we use the 450 K BeadChip to assess sex differences in DNA methylation from umbilical cord blood from boys and girls participating in a large epidemiologic cohort followed by the Center for the Health Assessment of Mothers and Children of Salinas (CHAMACOS) study. We use DCCs to account for white blood cell composition. In addition to interrogating DMPs, we apply the newly released ‘DMRcate’ methodology [14] to identify sex-associated DMRs in newborns.

## Methods

### Study population

The CHAMACOS study is a longitudinal birth cohort study of the effects of exposure to pesticides and environmental chemicals on the health and development of Mexican-American children living in the agricultural region of Salinas Valley, CA. Detailed description of the CHAMACOS cohort has previously been published [15, 16]. Briefly, 601 pregnant women were enrolled in 1999–2000 at community clinics and 527 liveborn singletons were born. Follow up visits occurred at regular intervals throughout childhood. For this analysis, we include the subset of subjects that had both 450 K BeadChip data and differential cell count analysis available at birth (*n* = 111). Mothers retained in the study subset had a mean age of 25.8 years (±5.1 SD) at time of delivery. Study protocols were approved by the University of California, Berkeley Committee for Protection of Human Subjects. Written informed consent was obtained from all mothers.

### Blood collection and processing

Cord blood was collected and stored in both heparin coated BD vacutainers (Becton, Dickinson and Company, Franklin Lakes, NJ) and vacutainers without anticoagulant at the same time. Blood clots from anticoagulant-free vacutainers were stored at −80 °C and used for isolation of DNA for DNA methylation analysis. Heparinized cord blood was used to prepare whole blood slides using the push-wedge blood smearing technique [17] and stored at −20 °C until staining for differential white blood cell count.

### DNA preparation

DNA isolation was performed using QIAamp DNA Blood Maxi Kits (Qiagen, Valencia, CA) according to manufacturer’s protocol with small, previously described modifications [18]. Following isolation, all samples were checked for DNA quality and quantity by Nanodrop 2000 Spectrophotometer (Thermo Scientific, Waltham, MA). Those with good quality (260/280 ratio exceeding 1.8) were normalized to a concentration of 50 ng/ul.

### 450 K BeadChip DNA methylation analysis

DNA samples were bisulfite converted using Zymo Bisulfite Conversion Kits (Zymo Research, Irvine, CA), whole genome amplified, enzymatically fragmented, purified, and applied to Illumina Infinium HumanMethylation450 BeadChips (Illumina, San Diego, CA) according to manufacturer protocol. Locations of samples from boys and girls were randomly assigned across assay wells, chips and plates to prevent any batch bias. 450 K BeadChips were handled by robotics and analyzed using the Illumina Hi-Scan system. DNA methylation was measured at 485,512 CpG sites.

Probe signal intensities were extracted by Illumina GenomeStudio software (version XXV2011.1, Methylation Module 1.9) methylation module and back subtracted. Systematic QA/QC was performed, including assessment of assay repeatability, batch effects using 38 technical replicates, and data quality established as previously described [19]. Samples were retained only if 95 % of sites assayed had detection *P* > 0.01. Color channel bias, batch effects and difference in Infinium chemistry were minimized by application of All Sample Mean Normalization (ASMN) algorithm [19], followed by Beta Mixture Quantile (BMIQ) normalization [20]. Sites with annotated probe SNPs and with common SNPs (minor allele frequency >5 %) within 50 bp of the target identified in the MXL (Mexican ancestry in Los Angeles, California) HapMap population were excluded from analysis (*n* = 49,748). Probes where 95 % of samples had detection *P* > 0.01 were also dropped (*n* = 460). Since our analysis was focused on CpG sites associated with sex, we excluded sites on the Y chromosome (*n* = 95) and X-chromosome cross-reactive probes (*n* = 29,233) identified by Chen and colleagues [7]. Remaining CpGs included 410,072 sites for analysis of sex. Methylation values at all sites were logit transformed to the M-value scale to better comply with modeling assumption [21].

### Differential cell counts

Whole blood smear slides were stained utilizing a DiffQuik^®^ staining kit, a modern commercial variant of the Romanovsky stain, a histological stain used to differentiate cells on a variety of smears and aspirates. This staining highlights cytoplasmic details and neurosecretory granules, which are utilized to characterize the differential white blood count. The staining kit is composed of a fixative (3:1 methanol: acetic acid solution), eosinophilic dye (xanthene dye), basophilic dye (dimethylene blue dye) and wash (deionized water). For consistency and to ensure the best results the slides were all fixed for 15 min at 23 °C (room temperature), stained in both the basophilic dye and eosinophilic dye for 5 s each and washed after each staining period to prevent the corruption of the dye.

Slides were scored for white blood cell type composition by Zeiss Axioplan light microscope with 100× oil immersion lens. Scoring was conducted at the perceived highest density of white blood cells using the standard battlement track scan method, which covers the entire width of a slide examination area. Counts for each of the five identifiable cell types (lymphocytes, monocytes, neutrophils, eosinophils, and basophils) were recorded by a dedicated mechanical counter. At least 100 cells were scored for each slide following validation of reproducibility by the repeated scoring of 5 sets of 100 cells from the same slide (CV ≤ 5 %).

### DMP analysis

Association between sex at birth and differential 450 K DNA methylation at individual CpGs was performed by linear regression, adjusting for DCC variables and analysis batch. This analysis was performed using R statistical computing software (v3.1.0) [22]. Although DCC estimates were not significantly associated with sex, we chose to include them in the model because likelihood ratio tests showed that including them improved model fit for more than 2000 of the CpG sites assessed by 450 K BeadChip. We also examined gestational age and subject birthweight as possible covariates since both have been shown to be associated with DNA methylation [23], and performed sensitivity analysis to assess their potential impact. However, neither was associated with child sex or contributed to improved model fit.

P-values were corrected for multiple testing using a Benjamini-Hochberg (BH) FDR threshold of 0.05 [24].

### Enrichment of annotated genomic features

Comparison of sex-DMP results to annotated function categories, including relation to genes(TSS1500, TSS200, 5′UTR, 1stExon, Body, 3′UTR, Intergenic) and CpG islands (Island, Shore, Shelf, Open Sea), was performed using UCSC Genome Browser annotations supplied by Illumina. A χ^2^ test of independence with 1° of freedom was used to determine whether there was evidence of enrichment among DMP results (*P* value < 0.05).

### DMR analysis

Identification of sex-associated DMRs was performed using the method described by Peters et al. [14] and implemented in the *DMRcate* Bioconductor R-package [25]. The approach begins by fitting a standard *limma* linear model to all CpG sites in parallel [26]. This model was parameterized identically to the DMP analysis with sex as the binary predictor of interest, adjusting for DCC variables and analysis batch. The CpG site test statistics were then smoothed by chromosome according to the *DMRcate* defaults, which employs a Gaussian kernel smoother with bandwidth λ = 1000 base pairs (bp) and scaling factor C = 2. The resulting kernel-weighted local model fit statistics were compared to modeled values using the method of Satterthwaite [27] to produce p-values that are adjusted for multiple testing using a BH FDR threshold of 0.05 [24]. Regions or DMRs were assigned by grouping FDR significant sites that are a maximum of λ bp from one another and contain at least two or more CpGs. Under this method, CpGs are collapsed into DMRs without considering the direction of the association with the predictor (i.e. sex). The minimum BH-adjusted p-value within a given DMR is taken as representative of the statistical inference for that region and the maximum fold change in methylation values (here on the M-value scale) summarizes the effect size.

### Gene ontology analysis

Gene ontology term enrichment analysis was performed by DAVID [28, 29], WebGestalt (WEB-based Gene SeT AnaLysis) [30], and ConsensusPathDB [31], using hypergeometric distribution to assess enrichment significance. Visualization of results and GO term categorization by semantic similarity dimension reduction was performed by REVIGO [32].

## Results

### Sex-associated differentially methylated positions in newborns

**Fig. 1:**
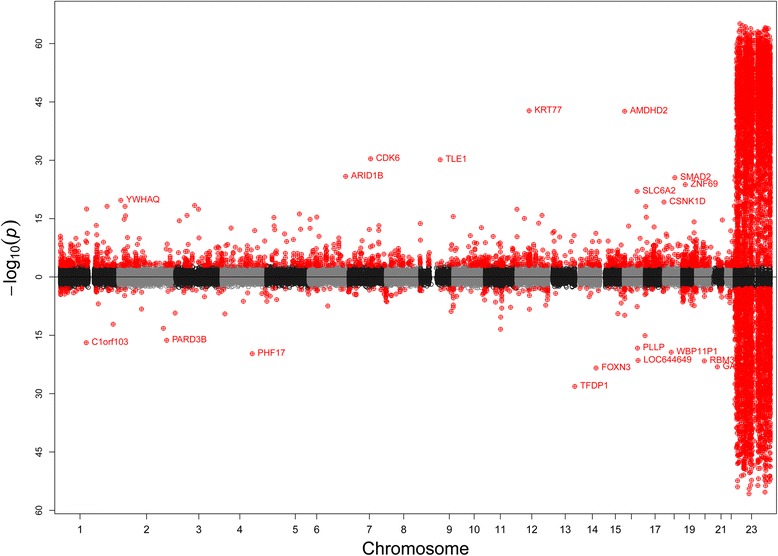
Manhattan plot for association between child sex and DNA methylation at all 450 K CpGs, adjusting for batch and cell composition by differential cell count (DCC). Associations where methylation was higher for girls relative to boys are plotted above the x-axis, while those with decreased methylation are plotted below. CpGs meeting FDR multiple testing threshold of (*P* < 0.05) shown in red

Analysis of DNA methylation differences between newborn boys and girls was performed by linear regression for 450 K BeadChip CpGs among subjects with DCC measurements (*n* = 111; 58 girls and 53 boys), adjusting for cell composition and batch (Table 1). After data cleaning, *n* = 410,072 CpGs were analyzed, which excluded sites previously reported to exhibit sex-chromosome specific cross-reactivity [7]. Resulting p-values were plotted by chromosome, with sites having higher methylation levels in girls compared to boys plotted above the x-axis and those with lower levels plotted below (Fig. 1). After adjustment for multiple testing (FDR *p* < 0.05), we identified 11,776 CpGs that differed significantly by sex in newborns (Table 2). Of those hits, the majority of sites had higher methylation in girls compared to boys (69.0 %). This trend was consistent on both the X chromosome (64.3 % of sites higher in girls) and in autosomes (82.8 %). While the majority of hits were found on the X chromosome (74.3 %), a substantial number were also identified on autosomes (3031 or 25.7 %; Table 2).

**Table 1:** Demographic characteristics of newborn CHAMACOS subjects, *N* = 111

	Boys, *N* = 53		Girls, *N* = 58		
	*N*	%	*N*	%	*P* Value*
Country of birth					0.18
Mexico	44	46.8	50	53.2	
United States	6	42.9	8	57.1	
Other	3	100	0	0	
Maternal age at delivery (years)					
18–24	26	55.3	21	44.7	0.54
25–29	16	43.2	21	56.8	
30–34	8	38.1	13	61.9	
35–45	3	50	3	50	
Family income					0.58
Below poverty threshold	32	50	32	50	
Above poverty threshold	21	44.7	26	55.3	
Maternal BMI (kg/m^2^)					0.31
Normal	24	51.1	23	48.9	
Overweight	14	37.8	23	62.2	
Obese	14	56	11	44	
Smoking during pregnancy					0.07
No	51	50.5	50	49.5	
Yes	2	20	8	80	
	Boys, *N* = 53		Girls, *N* = 58		
	Mean ± SD		Mean ± SD		*P* Value**
Gestational age (weeks)	39.1 ± 1.4		38.7 ± 2.0		0.35
Birthweight (Kg)	3.5 ± 0.5		3.4 ± 0.6		0.35
Blood cell composition (%)					
Lymphocytes	28.7 ± 4.5		29.6 ± 2.5		0.15
Monocytes	7.1 ± 1.9		6.8 ± 1.8		0.48
Neutrophils	60.5 ± 1.8		60.4 ± 2.9		0.53
Eosinophils	3.1 ± 1.2		2.8 ± 1.0		0.44
Basophils	0.2 ± 0.3		0.3 ± 0.5		0.27

*P value from χ2 test for independence

**P value from Mann-Whitney U test

**Table 2:** Summary of sex-associated DMPs

		Autosomes		X chromosome	
	All CpG sites	Count	%	Count	%
Hypermethylated in girls compared to boys	8,131	2,509	30.9	5,622	69.1
Hypomethylated in girls compared to boys	3,645	522	14.3	3,123	85.7
Total	11,776	3,031	25.7	8,745	74.3

Number of CpGs significantly hyper- and hypo-methylated in newborn girls compared to boys at FDR multiple testing threshold (q < 0.05), for all CpGs, and then stratified by autosomes and the X chromosome

**Fig. 2:**
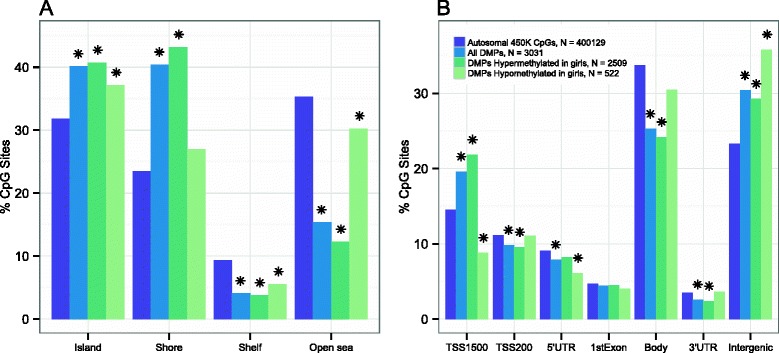
Percent of 450 K CpGs (purple), and percent of all (blue), hypermethylated (dark green), and hypomethylated (light green) autosomal differentially methylated positions (DMPs) associated with sex (**a & b**). These percentages are given by island functional categories (island, shore, shelf, and open sea) in **a**, and gene functional categories (within 1500 bp of a transcription start site (TSS), 200 bp of a TSS, a 5′ untranslated region (UTR), first exon, gene body, 3′UTR, and intergenic) in **b**. * indicates that the proportion of sites significantly altered compared to the coverage on the 450 K BeadChip (*P* < 0.05)

As differential hypermethylation is to be expected for girls due to X-inactivation [33–35], we focused characterization of results on autosomal sites showing sex differences (Table 3 and Additional file 1). Most of these were located in CpG shores, islands and open sea (40.4, 40.1, and 15.4 %, respectively) (Fig. 2 and Table 4). In comparison, shelf regions had the lowest percentage of hits (4.1 %). To assess whether the overrepresentation of hits in CpG islands and shores was due to the design of the 450 K BeadChip, we compared the number of hits in each functional category with the number of CpG sites included in the assay. Both shores and CpG islands were significantly overrepresented among all autosomal hits compared to the 450 K background (*χ*^2^ = 486.1, *P* < 0.01 and *χ*^2^ = 95.5, *P* < 0.01), while shelves and the open sea hits were underrepresented (each with *P* < 0.01). For CpG sites that were hypermethylated in girls compared to boys, we also observed overrepresentation in CpG islands and shores, and underrepresentation in shelf and open-sea locations (all *P* < 0.01). Sites that were hypomethylated in girls compared to boys were underrepresented in the open sea (30.3 %, *P* < 0.01) and shelves (5.6 %, *P* < 0.01). Hypomethylated sites were enriched at islands (*χ*^2^ = 6.53, P = 0.01), but did not deviate significantly from the 450 K representation of shores (*χ*^2^ = 3.42, P = 0.06).

**Table 3:** Results for the top 30 gene-annotated autosomal DMPs associated with sex in CHAMACOS newborns

ProbeID	Gene symbol(s)	Chromosome	Position^a^	β_Girl_ (M-value)	*P*-value	Girl mean (% methylation)	Boy mean (% methylation)
cg03691818	KRT77	12	53085038	2.38	1.84E-43	11.9	3.0
cg26921482	AMDHD2	16	2570283	1.72	2.43E-43	43.8	20.1
cg17743279	CDK6	7	92463268	1.84	4.04E-31	3.1	1.0
cg07852945	TLE1	9	84303915	0.93	7.30E-31	14.0	7.8
cg26355737	TFDP1	13	114292172	−0.95	7.57E-29	80.6	88.5
cg25568337	ARID1B	6	157098338	0.68	1.36E-26	21.2	14.6
cg05100634	SMAD2	18	45457604	1.77	2.91E-26	2.4	0.9
cg03608000	ZNF69	19	11998623	0.86	1.76E-24	5.5	3.1
cg02325951	FOXN3	14	89878619	−0.72	4.04E-24	62.4	73.0
cg17612569	GABPA;ATP5J	21	27107221	−3.07	7.86E-24	1.0	6.8
cg04874129	SLC6A2	16	55690873	0.75	9.82E-23	14.1	9.0
cg08906898	RBM39	20	34319899	−0.92	2.66E-22	87.2	92.6
cg04946709	LOC644649	16	59789030	−0.73	3.58E-22	70.1	78.8
cg02989351	YWHAQ	2	9770584	0.37	1.90E-20	16.5	13.2
cg12204423	PHF17	4	129732568	−0.82	1.94E-20	5.0	8.3
cg25304146	WBP11P1	18	30092971	−0.49	4.64E-20	57.6	64.9
cg22345911	CSNK1D	17	80231263	1.18	5.43E-20	5.2	2.7
cg01906879	GBE1	3	81811016	0.54	4.17E-19	8.5	6.0
cg06152526	PLLP	16	57290525	−0.62	5.25E-19	69.8	77.5
cg04190002	SHANK3	22	51113604	0.40	5.35E-19	33.8	27.9
cg06644124	ZNF281	1	200379083	0.50	6.63E-19	31.4	25.1
cg07628841	GPN1;CCDC121	2	27851430	0.30	7.09E-19	42.3	37.3
cg23001456	KIAA0664	17	2615074	0.86	7.47E-19	4.5	2.8
cg26213873	CTTNBP2NL	1	112939056	0.41	3.34E-18	8.6	6.6
cg25438440	CLDND1	3	98241168	0.55	3.72E-18	6.1	4.2
cg07816873	ERC1	12	1100472	0.54	3.88E-18	23.4	17.5
cg24016844	C1orf103	1	111506641	−0.43	1.28E-17	7.4	9.6
cg11841231	PARD3B	2	205543309	−0.68	5.39E-17	79.8	85.9
cg13323902	VTRNA1-1	5	140090859	0.53	6.23E-17	26.0	19.8
cg12900929	PRDM4	12	108154862	0.83	1.41E-16	11.2	6.9

Regression coefficients, β_girl_, are reported in M-value scale for the change in methylation of girls relative to boys. Girl and boy mean methylation levels are shown on the β value or % methylation

^a^Positions shown for hg19 (Genome Reference Consortium GRCh37) genome assembly

**Table 4:** DMPs by gene and CpG island annotation

		Sex DMPs		
	Autosomal 450 K	All DMPs	Hypermethylated	Hypomethylated
Island	127,488	1,216	1,022	194
Shore	93,981	1,225	1,084	141
Shelf	37,490	124	95	29
Open sea	141,170	466	308	158
Total	400,129	3,031	2,509	522
TSS1500	58,088	594	548	46
TSS200	44,647	298	240	58
5′UTR	36,279	239	207	32
1stExon	18,842	134	113	21
Body	135,011	766	607	159
3′UTR	14,073	79	60	19
Intergenic	93,189	921	734	187
Total	400,129	3,031	2,509	522

Counts of all, hypermethylated, and hypomethylated autosomal CpGs associated with sex in newborns by proximity to CpG island and gene features. Count of autosomal and all 450 K CpGs shown for reference

The 11,776 CpG hits differentially methylated between newborn boys and girls were found in 2250 unique genes, and 1430 (63.6 %) of these genes were located on autosomes. Many genes contained multiple significant sites, with an average of 4.7 CpGs per gene and a maximum of 114 CpGs. However, the largest portion of sex-associated autosomal hits (30.4 %) was located in intergenic regions and seen at lower than expected frequency in gene bodies (*P* < 0.01)(Fig. 2). Near gene transcription starting points (TSS200, 5′UTR, and first exons), all categories were either lower than 450 K CpG design frequencies or did not deviate from them significantly. Further upstream (TS1500), hits that were hypermethylated in girls were significantly enriched (*χ*^2^ = 108.5, *P* < 0.01) while those showing decreased methylation were underepresented (*χ*^2^ = 13.3, *P* < 0.01). At the end of genes (3′UTR), hits that had higher methylation for girls were underrepresented (2.4 %, *P* < 0.01), while hits having higher methylation for boys did not deviate from expected 450 K frequencies (3.6 %, *p* = 0.97).

Examining the autosomal genes containing sex-associated DMPs for enrichment of particular gene ontology (GO) terms identified 278 pathways that were significantly enriched (FDR *P* < 0.05 and at least 5 genes per GO term) (Table 5). These enriched GO terms fell into several broad categories including: 1) nervous system development, 2) behavior, 3) cellular development processes, and 4) cellular signaling and motility (Additional file 2).

**Table 5:** The top 30 differentially enriched gene ontology pathways among hits for sex in autosomal CpGs

			Genes		
GO category	Category name	Total	Changed	*P*-value	FDR
GO:0043005	Neuron projection	651	67	2.22E-05	0.00E + 00
GO:0007275	Multicellular organismal development	4621	353	9.06E-12	4.91E-09
GO:0097458	Neuron part	980	104	8.83E-11	8.47E-09
GO:0044767	Single-organism developmental process	5402	394	2.05E-10	1.24E-08
GO:0048856	Anatomical structure development	4828	359	2.43E-10	1.24E-08
GO:0007268	Synaptic transmission	692	78	1.50E-09	5.09E-08
GO:0048731	System development	4093	311	5.69E-10	1.54E-07
GO:0007270	Neuron-neuron synaptic transmission	129	26	3.30E-09	4.76E-07
GO:0007267	Cell-cell signaling	1192	115	3.51E-09	4.76E-07
GO:0043167	Ion binding	6038	422	2.02E-08	1.25E-06
GO:0007626	Locomotory behavior	186	30	1.25E-07	3.19E-06
GO:0032879	Regulation of localization	2010	168	4.15E-08	4.50E-06
GO:0044707	Single-multicellular organism process	6462	438	2.47E-07	5.04E-06
GO:0009653	Anatomical structure morphogenesis	2484	196	2.97E-07	5.05E-06
GO:0044765	Single-organism transport	3605	262	1.79E-06	2.36E-05
GO:0044763	Single-organism cellular process	11,949	739	1.85E-06	2.36E-05
GO:0009790	Embryo development	998	94	5.15E-07	4.66E-05
GO:1902578	Single-organism localization	3791	271	4.22E-06	4.79E-05
GO:0007399	Nervous system development	2053	171	4.97E-08	4.81E-05
GO:0065008	Regulation of biological quality	3239	236	4.77E-06	4.86E-05
GO:0051703	Intraspecies interaction between organisms	40	11	6.02E-06	5.58E-05
GO:0009887	Organ morphogenesis	918	87	9.65E-07	6.55E-05
GO:0048513	Organ development	2958	222	9.66E-07	6.55E-05
GO:0036477	Somatodendritic compartment	562	60	1.10E-06	8.50E-05
GO:0044459	Plasma membrane part	2279	174	1.90E-06	9.14E-05
GO:0016043	Cellular component organization	5410	366	1.68E-05	1.43E-04
GO:0030425	Dendrite	378	45	1.15E-06	1.70E-04
GO:0051705	Multi-organism behavior	79	15	2.27E-05	1.78E-04
GO:0005883	Neurofilament	9	6	2.54E-06	1.88E-04
GO:0035637	Multicellular organismal signaling	751	86	7.94E-08	2.00E-04

### Sex-associated differentially methylated regions in newborns

**Fig. 3:**
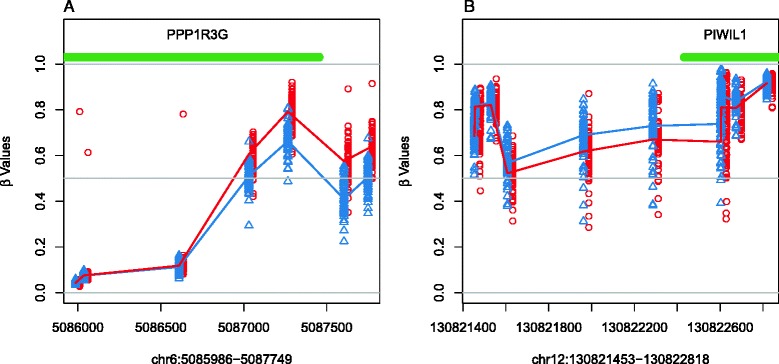
DNA methylation (β values) for CpG sites included in two top DMRs associated with child sex in newborns. One DMR (**a**) contains 7 CpG sites, is located on chromosome 6 and spans a 1763 bp region in the exon of *PPP1R3G* (chr6:5085986–5087749). The other (**b**) on chromosome 12 includes 8 CpGs over a 1365 bp region across the promoter and 1^st^ exon of *PIWIL1* (chr12:130821453–130822818). Girls are shown with red circles, boys with blue triangles, and median methylation per CpG by sex is shown by red and blue lines. Green lines show the genomic coordinates of exon regions for each gene shown

Additionally, identification of groups of CpGs with 450 K BeadChip methylation differences between newborn boys and girls was performed using the DMR-finding algorithm *DMRcate* [14, 25]. This approach identifies and ranks DMRs by Gaussian kernel smoothing of results from linear models for individual CpGs that were adjusted for cell composition and array batch (see Methods for details). A total of 3604 DMRs were significantly associated with sex in newborns after correcting for multiple testing (FDR *p* < 0.05; Table 6 and Additional files 3 and 4). These spanned 2608 genes and contained a total of 22,402 unique CpGs. The number of sites within the DMRs ranged from 2 to 99 CpGs, with 50 % of DMRs containing 5 or more CpGs and 25 % having 8 or more. Further, DMR length averaged 863.8 bp, and ranged from 3 to 16.5 kb. Figure 3 shows the DNA methylation levels for boys and girls at two example top DMRs. Figure 3a shows 7 CpG sites in a DMR that had higher methylation for girls in a region spanning the *PPP1R3G* transcription factor on chromosome 6. While Fig. 3b shows a 8 CpGs from a DMR with lower methylation among girls in the promoter of *PIWIL1*, which is an important gene for stem cell proliferation and inhibition of transposon migration [36, 37].

**Table 6:** Results for the top 30 gene-annotated autosomal DMRs associated with sex in CHAMACOS newborns

Gene symbol(s)	Chromosome	Start position^a^	End position^a^	# of probes	MaxFC for girls compared to boys	Minimum *P*-value
KRT77	12	53084709	53085323	4	0.09	1.34E-132
AMDHD2,ATP6V0C	16	2569911	2571449	9	0.24	7.17E-129
PPP1R3G	6	5085986	5087749	7	0.16	3.35E-78
CDK6	7	92461971	92464481	14	0.02	1.89E-65
TFDP1	13	114291977	114292740	10	−0.08	4.54E-57
CYP1A1	15	75018150	75019376	26	0.12	6.24E-57
C6orf174	6	127796287	127797286	7	0.12	2.64E-54
SMAD2	18	45456441	45458698	11	0.02	1.47E-51
ARID1B	6	157097800	157099375	9	0.07	6.59E-48
PEX10	1	2344089	2347015	26	0.07	6.53E-46
ATP5J,GABPA	21	27106793	27108257	11	−0.06	9.90E-45
SLC6A2	16	55689865	55691102	9	0.05	1.00E-43
A1BG,NCRNA00181	19	58861502	58862398	6	0.11	1.14E-43
PHF17	4	129731835	129733574	8	−0.03	2.69E-43
NUPL1	13	25874859	25876335	14	0.11	8.61E-42
PPFIA3	19	49636270	49636594	3	0.15	2.68E-40
ZNF69	19	11998457	11999148	11	0.03	4.58E-39
REM1,NCRNA00028	20	30071726	30073576	9	0.11	7.17E-39
YWHAQ	2	9770130	9771347	7	0.03	1.00E-37
LOC644649	16	59788728	59790180	7	−0.09	1.74E-35
FOXN3	14	89878584	89878733	5	−0.11	2.22E-35
PXDNL	8	52320944	52322341	9	0.07	7.31E-35
SHANK3	22	51112536	51114364	4	0.06	1.86E-34
SHANK2	11	70672365	70673256	11	−0.06	1.33E-33
RBM39	20	34319899	34319989	2	−0.06	1.74E-33
GIPC2	1	78511140	78512129	12	0.06	1.75E-33
CSNK1D	17	80230660	80232440	12	0.03	1.82E-33
NAPSA	19	50860534	50862121	8	−0.10	2.28E-33
FBXO47	17	37123638	37124558	9	0.08	2.94E-31
CCDC121,GPN1	2	27850964	27852231	14	0.05	5.71E-31

Max fold changes (FC) reported in M-value scale for the change in methylation of girls relative to boys

^a^Positions shown for hg19 (Genome Reference Consortium GRCh37) genome assembly

As with DMPs, the majority of sex-associated DMRs had higher methylation in girls compared to boys (75.8 %; Additional file 3: Table S1). This was true for both autosomes and sex chromosomes when considered individually, with 83.8 and 58.5 % of DMRs having higher methylation in girls, respectively. However, a greater total number of DMRs identified were located on autosomes (2471 or 68.6 %) compared to the X chromosome. Similarly, the 70.3 % of the genes covered by sex-associated DMRs were located on autosomes. Further, while the *DMRcate* method does not constrain all CpGs within a DMR to have the same direction of association with the predictor of interest, we found that the majority of DMRs had 100 % concordance across CpGs in the direction of effect with sex (Additional file 5).

Comparison of the individual site results (DMPs) with the DMR findings revealed that of the 11,776 CpG sites associated with sex in the DMP analysis, 9, 941 (84.4 %) were also included in a DMR. On autosomes, DMRs included 83.2 % of sites found as sex-associated DMPs. Conversely, the DMRs added 12,461 total sites (11,719 on autosomes) that had not been found by DMP analysis alone.

## Discussion

Here, we assessed methylation sex differences in newborns as determined by 450 K BeadChip. Using reliable DCC estimates, our results are the first reported EWAS analysis by sex at birth that adjusted for confounding by cell composition. To our knowledge, we are also the first study to assess regions of differential methylation associated with sex in addition to considering all CpG sites individually. We identified a large numbers of X-chromosome CpG sites with higher methylation in girls, which is most likely attributable to X-inactivation [33, 38]. Interestingly, we further demonstrated that a substantial number of autosomal sites and regions also appear hypermethylated in females (Fig. 1 and Table 2).

To assess the consistency of our findings with those of prior analyses, autosomal CpG sites identified as differentially methylated by sex in the current analysis were compared to hits from the three most similar published studies to date (Table 7) [8, 39, 40]. These studies differed from ours either in DNA methylation analysis platform (27 K in McCarthy et al. [18]) or in tissue type used (Xu et al. [39] in human prefrontal cortex and Hall et al. [40] in pancreatic isolates). Although the meta-analysis performed by McCarthy et al. included some studies in umbilical cord blood, most of the studies were performed in adult tissues. Each study found between 184 and 614 autosomal CpG sites that were differentially methylated in association with sex (total of *n* = 1192 unique sites across all three studies). Our results replicated 428 (35.9 %) of all hits, and 29.4–42.4 % by different studies. Further, among replicated sites we observed 98.5–100 % concordance in the direction of methylation differences. While there was substantial overlap between our autosomal sex-associated hits and these previously published results, 2603 or 85.9 % of our results are novel findings, some of which may be specific to the time point and tissue assessed (umbilical cord blood). Our larger number of hits is likely due to the increased coverage of the 450 K BeadChip. In fact, when considered as a percentage of the number of sites analyzed, we observed a comparable portion of autosomal hits to that found by McCarthy and colleagues using the 27 K platform (0.74 and 0.68 % respectively; *P* = 0.25).

**Table 7:** Comparison of CHAMACOS autosomal sex-associated CpG sites (*n* = 3031) with other published studies

Study	Study population	Tissue	Platform	Number of autosomal hits	Number of CHAMACOS hits replicated (%)	Percent of concordance in direction of methylation differences among replicated sites
McCarthy et al. 2014 [18]	Meta analysis of 76 studies (*n* = 6,795)	Multiple types	Illumina 27 K	184	54 (29.4 %)	100 %
Xu et al. 2014 [39]	46 Caucasian adults	postmortem prefrontal cortex	Illumina 450 K	614	260 (42.4 %)	98.50 %
Hall et al. 2014 [40]	87 Caucasian adults	pancreas	Illumina 450 K	470	176 (37.5 %)	100 %

Importantly, the autosomal methylation increases we observed were most concentrated in CpG islands and shores (Fig. 2a). As this trend was not evaluated in past studies, it should be explored and confirmed in additional datasets. Further, our findings that neurodevelopmental ontology terms were strongly enriched among our autosomal findings suggests that DNA methylation may contribute to differences in cognitive processes early in life. This is consistent with sex differences in brain development and rates of maturation that have previously been observed by magnetic resonance imaging in slightly older children (6–17 years of age) [41] and represent a possible regulatory mechanism requiring additional investigation.

Our autosomal hits included several genes already known to exhibit sex-specific functions. These included the male fertility and spermatogenesis related genes identified by McCarthy and colleagues (*DDX43*, *NUPL1*, *CRISP2*, *FIGNL1*, *SPESP1* and *SLC9A2*). One of our top hits showing increased methylation for girls (Table 3) included *SLC6A4,* Solute Carrier Family 6, that is involved in presynaptic reuptake of norepinephrine and has been implicated in several neurological disorders with sex-differences in prevalence [42–44]. Similarly, we observed novel sex differences in the *SHANK2* and *SHANK3* scaffolding protein genes that have been associated with autism spectrum disorders (Tables 3 and 6, Additional file 1) [45, 46]. Further, our hits included the homeobox containing transcription factor *EMX2*, Empty Spiracles Homeobox2, that is required for sexual differentiation and gonadal development [47] and we found to be hypermethylated among girls (Additional file 1).

The DMR analysis confirmed several trends observed by analyzing CpGs individually. In particular, DMR results again showed that girls tend to exhibit hypermethylation compared to boys. Also, many CpGs found to be autosomal DMPs were separately identified as being located within sex-associated DMRs. Besides confirming many of the findings in the DMP analysis, the application of DMR-finding substantially expanded the number of CpG sites considered significant. These results demonstrate that considering methylation over regions rather than single CpG sites may be a more effective way to identify differentially methylated sites and genes of interest.

## Conclusions

We confirmed and expanded previously identified trends in autosomal and X-chromosome methylation sex differences during a previously unstudied window in child development, immediately after birth, likely critical in establishing long term health. This strategy to assess epigenetic perturbation as near as possible to the prenatal period remains a high priority in light of the fetal origins of human disease hypothesis [48–51].

## Additional files

Additional file 1:
***Sex-associated autosomal DMPs.*** Results for all significant autosomal DMPs associated with sex in CHAMACOS newborns ranked by P value. (CSV 150 kb) Additional file 2:
***Visualization of enriched gene ontology categories.*** Gene ontology categories significantly enriched (PBH <0.05) in genes with sex-modified autosomal CpG sites. (PDF 30 kb) Additional file 3:
***Summary of sex-associated DMRs.*** Number of DMRs significantly hyper- and hypo-methylated in newborn girls compared to boys at FDR multiple testing threshold (q < 0.05), for all DMRs, and then stratified by autosomes and X chromosome. (XLSX 9 kb) Additional file 4:
***Sex-associated DMRs.*** Results for all significant DMRs associated with sex in CHAMACOS newborns ranked by P value. (CSV 449 kb) Additional file 5:
***Distribution of effect direction concordance within DMRs.*** Histogram of percent concordance of direction of sex-association for CpGs within identified DMRs. (PDF 9 kb)
