# Supplementary figures and images for: Sex differences in DNA methylation assessed by 450 K BeadChip in newborns
Source: BMC Genomics. 2015 Nov 9;16:911. doi: 10.1186/s12864-015-2034-y (PMC4640166; doi:10.1186/s12864-015-2034-y)

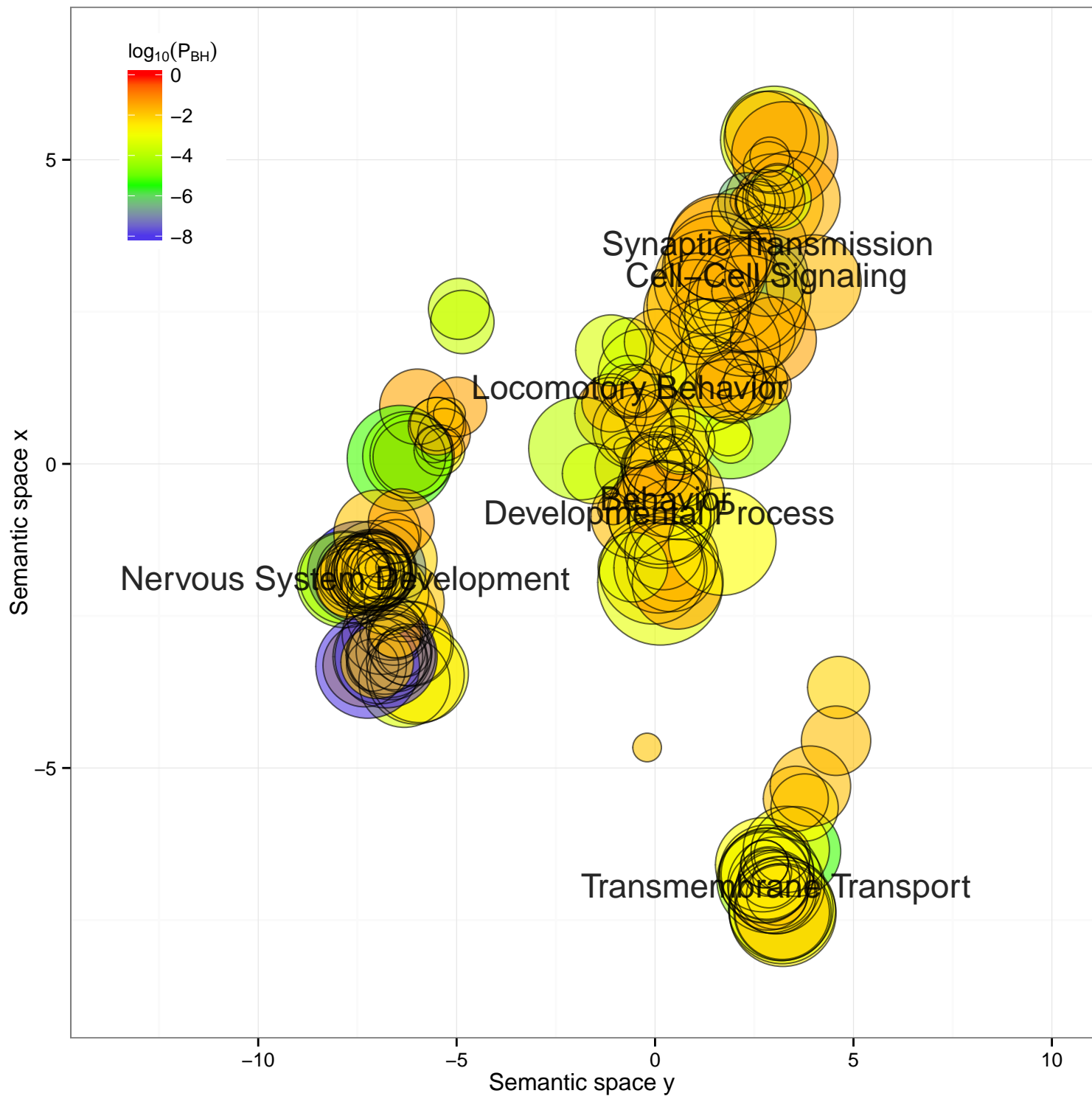

Supplement: Additional file 2: — Visualization of enriched gene ontology categories. Gene ontology categories significantly enriched (PBH <0.05) in genes with sex-modified autosomal CpG sites. (PDF 30 kb) [file 12864_2015_2034_MOESM2_ESM.pdf]

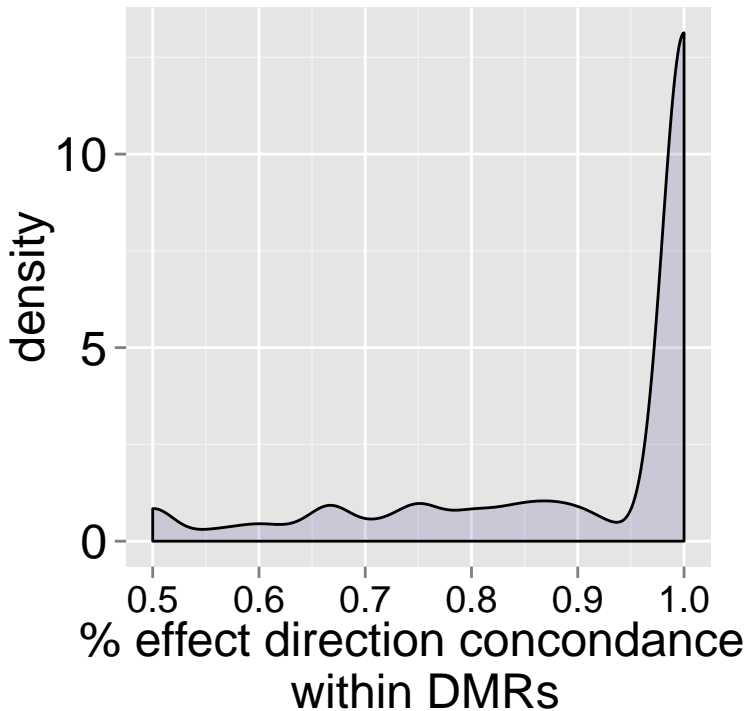

Supplement: Additional file 5: — Distribution of effect direction concordance within DMRs. Histogram of percent concordance of direction of sex-association for CpGs within identified DMRs. (PDF 9 kb) [file 12864_2015_2034_MOESM5_ESM.pdf]
